# Automatic learning of pre-miRNAs from different species

**DOI:** 10.1186/s12859-016-1036-3

**Published:** 2016-05-28

**Authors:** Ivani de O. N. Lopes, Alexander Schliep, André P. de L. F. de Carvalho

**Affiliations:** Empresa Brasileira de Pesquisa Agropecuária, Embrapa Soja, Caixa Postal 231, Londrina-PR, 86001-970 CEP Brasil; Department of Computer Science, Rutgers University, 110 Frelinghuysen Road, Piscataway, 08854 NJ USA; Instituto de Ciências Matemáticas e de Computação, Avenida Trabalhador são-carlense, 400 - Centro, São Carlos SP, Brasil

## Abstract

**Background:**

Discovery of microRNAs (miRNAs) relies on predictive models for characteristic features from miRNA precursors (pre-miRNAs). The short length of miRNA genes and the lack of pronounced sequence features complicate this task. To accommodate the peculiarities of plant and animal miRNAs systems, tools for both systems have evolved differently. However, these tools are biased towards the species for which they were primarily developed and, consequently, their predictive performance on data sets from other species of the same kingdom might be lower. While these biases are intrinsic to the species, their characterization can lead to computational approaches capable of diminishing their negative effect on the accuracy of pre-miRNAs predictive models. We investigate in this study how 45 predictive models induced for data sets from 45 species, distributed in eight subphyla/classes, perform when applied to a species different from the species used in its induction.

**Results:**

Our computational experiments show that the separability of pre-miRNAs and pseudo pre-miRNAs instances is species-dependent and no feature set performs well for all species, even within the same subphylum/class. Mitigating this species dependency, we show that an ensemble of classifiers reduced the classification errors for all 45 species. As the ensemble members were obtained using meaningful, and yet computationally viable feature sets, the ensembles also have a lower computational cost than individual classifiers that rely on energy stability parameters, which are of prohibitive computational cost in large scale applications.

**Conclusion:**

In this study, the combination of multiple pre-miRNAs feature sets and multiple learning biases enhanced the predictive accuracy of pre-miRNAs classifiers of 45 species. This is certainly a promising approach to be incorporated in miRNA discovery tools towards more accurate and less species-dependent tools.

The material to reproduce the results from this paper can be downloaded from http://dx.doi.org/10.5281/zenodo.49754.

**Electronic supplementary material:**

The online version of this article (doi:10.1186/s12859-016-1036-3) contains supplementary material, which is available to authorized users.

## Background

MicroRNAs (miRNAs) constitute one of the most widely-studied class of endogenous small (approx. 22 nucleotides) non-coding RNAs genes, due to their regulatory role in post-transcriptional gene regulation in animals, plants and fungi [1, 2]. The miRNAs biogenesis involves the participation of several enzymes, which depend on the origin (e.g. intergenic or intronic miRNAs) and on the kingdom of the species. However, all miRNAs are processed from long primary miRNA transcripts (pri-miRNAs), which are processed to hairpin-shaped intermediates (pre-miRNAs) and, subsequently, to the double strand RNA miRNA:miRNA* and a terminal loop. The miRNA* strand is the reverse complement of the functional miRNA, which usually degrades after being unwound by the action of specific enzymes. In the cytoplasm of animal and plant cells, the mature miRNA enters in the RNA-induced silencing complex (RISC) to silence target messenger RNAs (tmRNAs) by partial or near-perfect antisense complementarity. Partial antisense complementarity inhibits the translation of tmRNAs, whereas the later causes the degradation of tmRNAs. Reviews on biogenesis, diversification and evolution of miRNAs can be obtained at [2–4].

RNAseq methods, followed by computational analysis, became the *de facto* approach for miRNA discovery [4]. These methods, also called deep sequencing of the transcriptome, can reveal the identities of most RNA species inside a cell, providing tens to hundreds of millions of sequence reads [5]. These reads provide both the sequence and the frequency of RNA molecules present in a cell. When applied to detect miRNAs, the RNA material is isolated through a procedure of size selection, such that only small reads (approx. 25 nt long) are sequenced [5]. The computational challenge consists in distinguishing miRNAs from other small RNA (sRNA) types and degradation products [4, 6].

The challenge of building a multi-species miRNA prediction tool is reflected in the wide range of sensitivities estimated for eight deep sequencing miRNA prediction tools, when they were applied to data sets from *H. sapiens*, *G. Gallus* and *C. elegans* [7]. The sensitivity ranges varied between 24 % and 38 %. For example, the sensitivity of the tool with the highest average sensitivity (68 %) varied between 55 % (*H. sapiens*) and 78 % (*G. Gallus*) and the sensitivity of the tool with lowest average sensitivity (15 %) varied between 0 % (*H. sapiens*) and 25 % (*C. elegans*). The species bias is also present in the analysis performed with miRDeep2 [8], a newer version of miRDeep [6], which incorporated additional features to increase the detection of known and novel miRNAs in all animal major clades. Even though the average sensitivity of miRDeep2 (80 %) has clearly increased compared to its first version, it still varies depending on the species from 71 % (Sea squirt) to 90 % (Anemone). In order to identify the source of these variabilities, it is imperative to explore how the main factors involved in the development of such computational tools vary throughout species.

As miRNAs are processed from hairpin regions, computational tools to predict miRNAs from RNA-seq libraries include at least four steps: pre-processing; read mapping to a reference genome; detection of energetically stable hairpins in the genomic region surrounding the mapped read and; detection of miRNAs biogenesis ‘signature’. The latter is derived from the abundance and from the distribution of the reads across the hairpin and is fundamental to reduce false detections, since the hairpin shape structure is a necessary but not sufficient condition to process miRNA. Three criteria have been used as evidence of miRNAs biogenesis: a) the frequency of the mature strand is higher than the frequencies of the corresponding star and loop strands; b) the positions of the Drosha and Dicer cleavage sites in the 5’ ends of the putative miRNA and miRNA* are nearly uniform and; c) the putative miRNA and miRNA* sequences align in the hairpin keeping approximately 2 nt overhang in the 3’ end [4]. Nevertheless, the hairpin analysis is possibly the most critic step affecting negatively the sensitivity of the tools, since the biogenesis signature analysis is performed either after the selection of the energetically most favorable hairpin containing the mapped read stack (e.g. as in miRanalyzer [9]) or simultaneously, where the distribution of the reads in the putative hairpin and hairpin features are considered (as in miRDeep2 [8]). Variants of those approaches have also been proposed in the literature. NoraDesk [10] was the first method to incorporate structural energy based features and read coverage information to increase the detection accuracy of small ncRNAs, including miRNAs, whereas miReader [11] relies only on hybridization patterns of the reads to detect miRNAs.

The hairpin analysis has been performed mostly through machine learning based predictive models. To obtain these models, a feature set (feature vector) describing sequence and/or structural aspects of pre-miRNAs sequences (+) and hairpin like (-) sequences is extracted to create a training data set, which is subsequently fed to a machine learning algorithm. An investigation on human pre-miRNAs classifiers indicated that the feature set, instead of the learning algorithm, had the major effect in the classification accuracy of the induced models [12]. However, the relevance of those features for the correct classification of pre-miRNAs from other species remained an open question.

Since miRNA systems in plants and animals differ substantially [4], computational tools for plant and animal miRNAs discovery have been developed separately (e.g. [9, 13]). However, in practice, even instances of species from the same kingdom apparently diverge substantially regarding their intrinsic and extrinsic features. Therefore, in order to develop miRNA discovery tools robust to species-specific differences, a first step is to determine if a unique feature set can capture the diversity of pre-miRNAs throughout species. Moreover, it is important to establish boundaries of the applicability of cross-species miRNAs predictive models, since the relevance of any tool depends on its ability to detect the miRNAs present in the data set under analysis. Another important aspect is the computational cost of extracting a feature set, since this cost can be prohibitive for some distinct pre-miRNAs features (e.g. energy stability parameters) if they are to be computed for millions of hairpins. These issues were addressed in this study, considering eight feature sets investigated in [12], three learning algorithms and 45 species representing eight subphyla/classes.

Our experimental results showed that the classification complexity of pre-miRNAs is species-dependent, albeit some feature sets and learning algorithms were more likely to maximize the predictive accuracy of pre-miRNAs classifiers for most species (first subsection of the Results and discussions section). To interpret this dependency, we analyzed how relevant the features extracted from instances of one species are for the classification of instances of other species (in the following subsections). This analysis indicated that pre-miRNAs classifiers restricted to predict instances of species from the same subphylum of the species used on its induction (training species), instead of the same kingdom, are more likely to achieve higher accuracies. Nevertheless, our results also showed that ensembles of classifiers using computationally inexpensive feature sets performed well even if the subphylum of the training species disagrees. The ensemble approach has the potential to extend the applicability of pre-miRNA predictive models to a broader number of species, while keeping the computational cost close to that of single classifiers.

## Methods

### Experimental design

The analysis carried out in this study was based on the accuracy of classifiers obtained in two steps: (1) create pre-miRNA data sets and (2) induce and test classifiers for classification of pre-miRNAs. In the step (1), for each species, 30 sequences from each class were randomly sampled from the pre-processed positive and negative sets to compose the test sets. From the remaining sequences, 60 sequences from each class were randomly sampled to construct the training set. Afterwards, all features were extracted from each sequence. This first step was repeated 10 times. As these data sets were built by species, they are also referred as training and test species. In the step (2), instances from all test sets were classified by the classifiers obtained with the training data built in the step (1). The accuracy of these classifiers were analyzed under the two-way analysis of variance (anova) Eqs. 1 and 2.

The sizes of the training and test sets were, respectively, 2/3 and 1/3 of the smallest number of positive non-redundant sequences, shown in the Additional file 1. Once training and test sequence sets had been randomly sampled, all features were extracted. Therefore, the data sets of feature vectors diverged only by the feature composition, which can be geometrically seen as different subspaces of the unknown space of the pre-miRNAs features. By fixing the sizes of training and test sets, we reduced the sources of random variations, i.e., variations that cannot be assigned to a main factor. Moreover, since our main goal was to study the effect of the training species (*S*) in the predictive accuracy of pre-miRNAs classifiers, we considered the effects of the classification algorithm and the feature set in a unique factor, represented here by *M*. Therefore, considering three algorithms and eight feature sets, the number of levels of the factor *M* is 24 (or 3 ×8).

#### Anova 1: M × S

The first analysis was performed to study the relationship between the factors *M* and *S* (*M*×*S*) in order to identify the levels of *M* that led to higher predictive accuracies for each species. For such, we considered the Eq. 1, where the accuracies were estimated considering the same training and test species.

1 $$ A_{ilk}=\mu + M_{l} + S_{i} + MS_{li} + {R_{k}}+{e}_{ilk},  $$

such that: *l*=1,…,24 indexes the classifiers, *i*=1,…,45 indexes the species, *k*=1,…,10 indexes the repetition, *A*_*ilk*_= accuracy of the classifier *l*, obtained with the training species *i* in the repetition *k*, *μ*= overall mean accuracy, *M*_*l*_= effect of the classifier *l*, *S*_*i*_= effect of the species *i*, *MS*_*li*_= interaction between the effects of the classifier *l* and the species *i*, and *R*_*k*_=effect of repetition k, blocking factor; *e*_*lik*_= random error, or part of *A*_*lik*_ that could not be assigned to the classifier *l*, the species *i* and the repetition *k*; *e*∼*N*(0,*σ*^2^).

#### Anova 2: cross-species classifiers

To investigate the suitability of instances from one species to build pre-miRNAs predictive models for other species, we fixed a classifier *l*, *l*=1,…, 24, and varied the training and test species. The accuracies were analyzed according to Eq. 2:

2 $$ A_{lijk}=\mu + M_{li} + T_{j} + MT_{lij} + {R_{k}}+{e}_{lijk},  $$

such that: *l* indexes one out the 24 classifiers, *i,j*=1,…,45 indexes training and test species, *k*=1,…,10 indexes the repetition, *A*_*lijk*_= accuracy of the classifier *l*, obtained with data from the species *i*, in predicting the classes of instances from the species *j* in repetition *k*, *μ*= overall mean accuracy, *M*_*li*_= effect of a species *i*, *T*_*j*_= effect of the species *j*, *MT*_*lij*_= effect of the interactions model species *i* and test species *j*, and *R*_*k*_=effect of repetition k, blocking factor; *e*_*lijk*_= random error, or part of *A*_*lijk*_ that could not be assigned to the species *i*, the test species *j* in the repetition *k*; *e*∼*N*(0,*σ*^2^).

#### Clustering algorithm

The Eqs. 1 and 2 are particularly useful to estimate the variance of random errors (*σ*^2^). Once this variance is known, we can decide how typical the variances estimated from the controlled factors (e.g. *M*, *S* and *MS*) are, compared to *σ*^2^, using the *p*-value obtained from the *F*-test. In this work, significant *p*-values were lower or equal to 0.05 (*p*≤0.05). Since significant *p*-values of *F*-test on a factor only supports the inference that at least two levels of that factor had different average effects, we applied a clustering algorithm due to Scott and Knott [14] to identify the levels of each factor in Eqs. 1 and 2 that led to non-significantly different accuracies using the R package ScottKnott [15].

### Data sets

#### Positive sequences

To construct positive data sets, we downloaded all pre-miRNAs from miRBase release 20. This release contains 24,521 miRNA loci from 206 species, processed to produce 30,424 mature miRNA products [16]. However, only 65 species had at least 100 pre-miRNAs. From these 65 species, 48 had at least 90 non-redundant sequences (see criterion in the pre-processing subsection). Based on the availability of sequences that could be used to generate negative examples, positive sequences from only 45 species were considered. The identification of these species per phylum/division, subphylum/class, the acronyms used in their identification, the amount of available and non-redundant pre-miRNAs, the mean and the standard deviation of their sequence length are shown in Additional file 1.

#### Negative sequences

Negative data sets were constructed from a pool of 1,000 pseudo hairpins per species. These pseudo hairpins were excised from Protein Coding Sequences (CDS) or pseudo gene sequences, downloaded from the repositories Metazome v3.0, Phytozome v9.0 or NCBI, as detailed in the Additional file 2. The excision points were randomly chosen in the interval [0,*L*−*l*_*pse*_−100], where *L* was the sequence length of the CDS or pseudo gene and *l*_*pse*_ was the length of the excised sequence. The number of pseudo hairpins of length *l*_*pse*_ were determined in accordance with the length distribution of the available pre-miRNAs from each species. Afterwards, the excised sequence was evaluated for the resemblance with real pre-miRNAs. Sequences that passed the criteria described in the items 1 to 4 below were stored as pseudo hairpins, and those that failed any of these criteria were discarded. These criteria were:

1. fold-back structure;
2. *bp*≥18, *bp* = base pairing;
3. *Q*_*seq*_≥0.9,*Q*_*seq*_ = sequence entropy;
4. Minimum Free Energy of folding (*MFE*) rules:
  $MFE_{l_{pse}} \le -10.0 $, if *l*_*pse*_<70
  $MFE_{l_{pse}} \le -18.0 $, if 70<*l*_*pse*_≤100
  $MFE_{l_{pse}} \le -25.0 $, if *l*_*pse*_>100.

*Q*_*seq*_ was used to filter out meaningless sequences, since genomic sequences are usually contiguously padded with "N" characters and the three *MFE* rules were applied to accommodated the correlation between *MFE* and *L* that occurs in pre-miRNAs.

#### Pre-processing

Genes in a miRNA family can have sequence identity of 65 % or higher [17]. Since the number of miRNA families is relatively small compared to the number of positive examples available, redundancy removal is an important pre-processing procedure to avoid overfitted predictive models. We used dnaclust [18] to remove redundant sequences, prior to the sampling of examples to compose training and test sets. With dnaclust, sequences in positive sets of each species were clustered such that the similarity between sequences within a cluster were at least 80 %. Afterwards, one sequence from each cluster was randomly sampled to construct the positive non-redundant sets. The same pre-processing procedure was applied to the sets of negative sequences. As detailed in Additional file 2, 15 or less sequences were removed from 35 out of 45 negative sequence sets. The relatively lower number of redundant pseudo hairpins in those sets, compared to pre-miRNAs sequence sets, is due to the random choice of the starting position of the pseudo hairpin excision. However, at least 35 redundant pseudo hairpins were removed from the other 10 sequence sets.

### Feature sets

The eight features sets primarily studied in this investigation were extensively evaluated on human sets by Lopes et al. [12]. Here, these feature sets are referred by the same notation (FS _*i*_,*i*∈{1,..,7} and SELECT). Table 1 presents the features that compose each feature set, along with references of computational pipelines where they have been used. Although detailed descriptions of these features can be found in the references cited in Table 1 or elsewhere, we provide next a short description of these features regarding four major categories: sequence composition features, structure based features, sequence-structure based features, thermodynamic features and probabilistic properties. Their representation in Table 1 are within parentheses in the text below.

**Table 1:** Feature set composition, dimension, literature reference

Feature	Feature set							
	FS_1_	FS_2_	FS_3_	FS_4_	FS _5_	FS_6_	FS_7_	Select
Di-nucleotide frequencies (*XY, X,Y*∈{*A,C,U,G*})	x							
*%* *G*+*C*	x	x					x	
Maximal length of the amino acid string without stop codons (*orf*)							x	
Percentage of low complexity regions (*dm*)							x	
Triplets				x		x		
Stacking triplets (*X*_(((_,*X*∈{*A,C,G,U*})							x	
Motifs (*s* *s*−substrings)					x			
Minimum free energy of folding (*MFE*)						x		
Randfold (*p*)						x		
Normalized MFE (*dG*)	x	x	x				x	x
MFE index 1 (*M* *F* *E* *I*_1_)	x	x	x				x	x
MFE index 2 (*M* *F* *E* *I*_2_)	x	x	x				x	x
MFE index 3 (*M* *F* *E* *I*_3_)	x	x					x	x
MFE index 4 (*M* *F* *E* *I*_4_)	x	x					x	
Normalized Ensemble Free Energy (*NEFE*)	x	x					x	x
Normalized difference (*M* *F* *E*−*E* *F* *E*) (*Diff*)	x	x					x	x
Frequency of the MFE structure (*Freq*)	x							
Normalized base-pairing propensity (*dP*)	x		x					
Normalized Shannon entropy (*dQ*)	x	x	x				x	x
Structural diversity (*Diversity*)	x	x					x	
Normalized base-pair distance (*dD*)	x		x					
Average base pairs per stem (Avg_Bp_Stem)	x	x					x	
Normalized A-U pairs counts (|*A*−*U*|/*L*)	x	x					x	
Normalized G-C pairs counts (|*G*−*C*|/*L*)	x	x					x	x
Normalized G-U pairs counts (|*G*−*U*|/*L*)	x	x					x	x
Content of A-U pairs per stem (*%*(*A*−*U*)/*s* *t* *e* *m* *s*)	x	x					x	
Content of G-C pairs per stem (*%*(*G*−*C*)/*s* *t* *e* *m* *s*)	x	x					x	
Content of G-U pairs per stem (*%*(*G*−*U*)/*s* *t* *e* *m* *s*)	x	x					x	x
Cumulative size of internal loops (*loops*)							x	
Structure entropy (*dS*)	x	x					x	x
Normalized structure entropy (*d* *S*/*L*)	x	x					x	x
Structure enthalpy (*dH*)	x							
Normalized structure enthalpy (*d* *H*/*L*)	x							
Melting energy of the structure	x							
Normalized melting energy of the structure	x							
Topological descriptor (dF)	x	x	x				x	x
Normalized variants (*zG*, *zP* and *zQ*)	x							
Normalized variants (*zD*)	x	x					x	
Normalized variants (*zF*)	x							
Dimension	48	21	7	32	1300	34	28	13
Reference	[21]	[21]	[33]	[23]	[34]	[35]	[19]	[12]

#### Sequence composition features

This category includes the dinucleotides contents (*%XY, X, Y*∈{*A,C,U,G*}) and the *G*+*C* or *A*+*G* contents (*%G*+*C* or *%A*+*G*), the maximal length of the amino acid string without stop codons (*orf*) and the percentage of low complexity regions (*dm*) in the sequence [19]. The first two groups of features are more intuitive, whereas the last two features may help to distinguish protein coding sequences from ncRNAs, since *%LCRs* are defined as short amino acid motifs or regions that contain repeats of single amino acids [20].

#### Structure based features

The predicted secondary structure is the intra-molecular accommodation demanding the Minimum Free Energy of folding (*MFE* or *G*). Some *MFE* variants have been proposed to correct the bias towards sequence length (*dG*), *%G*+*C* (*MFEI*_1_) and structural complexity (*MFEI*_2_,*MFEI*_3_ and *MFEI*_4_). In vivo, an RNA molecule commonly exists in an assembly of structures. The distribution of these structures can be modeled by a Boltzmann distribution of free energy and the probabilities of these structures are used to compute the Normalized Ensemble Free Energy (*NEFE*), the normalized difference (*Diff*=(*MFE*−*EFE*)/*L*) and the frequency of the *MFE* structure (*Freq*) [21]. Since pre-miRNAs are typically energetically more stable [22], the *MFE* and its variants are important feature for pre-miRNA prediction.

The minimum number of base-pairings (bp) in the secondary structure of pre-miRNAs is approximately 18 bp [23]. The normalized base-pairing propensity (*dP*) and the average base pairs per stem (*Avg*_*Bp*_*Stem*) indicates the occurrence of this pattern in a stem-loop structure, whereas the base-pair distance and its normalized version (*Diversity* and *dD*=*Diversity*/*L*) inform the structural diversity.

More complex patterns of the secondary structure are captured by the topological descriptor (*dF*), the normalized Shannon entropy (*dQ*) and the cumulative size of internal loops found in the secondary structure (*loops*). *dF* is the second eigenvalue of the Laplacian matrix of the graph representation of the secondary structure where, bulges, loops, and other related measures are the vertices and the stems are the edges. The parameter *dQ* characterizes the base-pairing probability distribution per base in a sequence, represented as a chaotic dynamical system [24]. Since the local dominance of a single structure within the Boltzmann distribution of alternative secondary structures is strongly correlated with the reliability of the *MFE* structure, *dQ* is a measure of well-definedness for the structure [25]. In addition, well-defined structures have lower Shannon entropy as compared to structures with many alternative competing base pairs. The last parameter, *loops*, has been proposed as a distinguishing feature for pre-miRNA prediction [19], since pre-miRNAs usually have smaller internal loops.

#### Sequence-structure based features

Features in this category include the sequence nucleotide information and its state in the predicted secondary structure. The abundance of Watson-Crick base-pairings A-U, C-G and G-U in the secondary structure was considered according to two normalization criteria: the sequence length *L* (|*A*−*U*|/*L*,|*C*−*G*|/*L*, | *G*−*U*|/*L*) and the number of stems on the secondary structure (|*A*−*U*|/*n*_*stems*,|*C*−*G*|/*n*_*stems*,|*G*−*U*|/*n*_*stems*). Another group of features accounts for the occurrence of certain patterns in the sequence and structure level. The triplets *s*_*l*_*s*_*x*_*s*_*r*_ represent the normalized frequency of three contiguous states ({ paired=*`*(^′^,unpaired=*`*.^′^}) in the secondary structure, where *s*_*x*_ is the state of a fixed middle nucleotide ({*A,C,G,U*}) and *s*_*l*_ and *s*_*r*_ are the states of *x*’s left and right neighbors. An extension of the triplets are the sequence-structure motifs. The relative occurrence of a motif is computed from the string obtained by padding the original sequence with the corresponding state of each nucleotide in the secondary structure, distinguishing left and right pairings.

#### Thermodynamic features

Thermodynamic features are structure entropy (*dS* and *dS*/*L*), structure enthalpy (*dH* and *dH*/*L*) and melting temperature (*T*_*m*_). The latter is estimated assuming that the sequence either folds or do not fold. Thus, the estimated *T*_*m*_ is the temperature where the fold-back or hairpin-like structure disrupts. *T*_*m*_ is related with the enthalpy and the entropy by the equation *T*_*m*_=*ΔH*/*ΔS*, where *Δ* represents variation.

#### Probabilistic properties

Probabilistic properties refer to parameters that measure the stability (or variation) of certain features when computed from a sequence and from its randomized (shuffled) versions [22]. Since real pre-miRNAs are energetically more stable than pseudo-hairpins, the differences between the *MFE* of a real pre-miRNAs and the mean *MFE* of its shuffled sequences (*MFE*_*shuf*_) are expected to be neglectable. Two different formulas to capture the energy stability have been proposed. The first, *zG* [24], is the difference between *MFE* and *MFE*_*shuf*_, in unities of standard deviation (*SD*_*shuf*_). The second, *p* (randfold) [22, 26], is the relative frequency by which *MFE*_*shuf*_ was lower than *MFE*. Similarly to the computation of *zG*, the *z*-variants of *dP*, *dQ*, *dD* and *dF* were also assessed and represented as *zP*, *zQ*, *zD* and *zF* [24].

### Learning algorithms

The learning algorithms used in this work were Support Vector Machines (SVMs), Random Forest (RF) and J48. These algorithms have different learning biases, which is important for the present work, since learning biases may favor a feature set over others. SVMs and RFs are the algorithms most frequently used for pre-miRNA classification and J48 was chosen because of its simplicity and interpretability.

J48 implements the well known C4.5 algorithm [27]. As one of the most popular algorithm based on the divide-and-conquer paradigm, C4.5 recursively divides the training set into two or more smaller subsets, in order to maximize the information entropy. The J48 implementation builds pruned or unpruned decision trees from a set of labeled training data. We used RWeka [28], an R interface of Weka [29], with the default parameter values. RWeka induces pruned decision trees from a data set.

To train SVMs, we used a Python interface for the library LIBSVM 3.12 [30]. This interface implements the C-SVM algorithm using the RBF kernel. The kernel parameters *γ* and *C* were tuned by 5-fold cross validation (CV) over the grid (*C*;*γ*) = (2 ^−5^,2^−3^,…,2^15^; 2 ^−15^,2^−13^,…,2^3^). The pair (*C*;*γ*) that led to the highest CV predictive accuracy in the training subsets was used to train the SVMs using the whole training set. The resulting classifier was applied to classify the instances from the corresponding test set.

RF ensembles were induced over the grid (30, 40, 50, 60, 70, 80, 90, 100, 150, 250, 350, 450) ×[ (0.5, 0.75, 1, 1.25, 1.5)*$\sqrt {d}$ ], representing respectively the number of trees and the number of features. The value $\sqrt {d}$ is the default number of features tried in each node split, where *d* is the dimension of the feature space or the number of features in the feature set. We chose the ensemble with the lowest generalization error over the grid, according to the training set, and applied it to classify the instances of the corresponding test set. The ensembles were obtained using the *randomForest* R package [31] in an *in house* R script.

#### Ensembles and other feature sets

In addition to the predictive accuracy, the applicability of any pre-miRNA classifier to larger data sets may be limited by the computational time necessary to compute the feature set representation of each pre-miRNA candidate. To increase the predictive accuracy while keeping the computational cost under feasible limits, subsets of the existing features sets, removing features computed from shuffled sequences, were employed to construct ensemble of classifiers. These subsets were named Ss1 and Ss7, such that: *Ss*1=FS_1_−{*zG,zP,zQ,zD,zF*} and *Ss*7={*orf*,*%LCRs,loops,A*_(((_,*C*_(((_,*G*_(((_,*U*_(((_}. Ss1 features measure the largest variety of pre-miRNA characteristics, whereas Ss7 combine features widely used in pre-miRNA classification (*A*_(((_,*C*_(((_,*G*_(((_,*U*_(((_) with three features introduced in pre-miRNA classification in [19]. The first subset was evaluated individually, and combined with the latter (Hyb_17_=*Ss*7∪*Ss*1). The subset Ss7 was also combined with the feature sets FS_3_ (Hyb_37_=FS_3_∪Ss7) and SELECT (Hyb_*S*7_=Ss7∪SELECT). The prefix Hyb is used to represent these ‘hybrid’ feature sets.

An ensemble of classifiers combine the predictions from a set of single classifiers. The ensembles used in this study are described in Table 2, along with all other classifiers investigated. The computational time for the extraction of the feature sets used in the ensembles are close to the time spent to extract the feature set SELECT and presented in [12]. As shown in this table, the final prediction of the ensembles were defined by majority vote (ensemble Emv) and by weighted vote (ensemble Ewv). In the first approach, the class predicted by the majority of the classifiers is the ensemble class prediction. In the weighted approach, the vote from each classifier was weighted by its predictive accuracy in the training sets. Ties were resolved by random choice.

**Table 2:** Definition of all 44 classification models compared in this work, according to feature sets and learning algorithms. M_*ij*_ is the classifier induced with the feature set *i* and algorithm *j*, *i*=1,…,12 and *j*=1,2,3, and *w*_*ij*_ is the cross-validation accuracy of the classifier M_*ij*_. $\hat {M}_{ij}$ is the predicted class by $\text {M}_{ij},\hat {M}_{ij} \in \{-1,1\}$. Emv=Ensemble majority votes, Ewv=Ensemble weighted votes

	1. SVMs	2. RF	3. J48
1. FS_1_	M_11_	M_12_	M_13_
2. FS_2_	M_21_	M_22_	M_23_
3. FS_6_	M_31_	M_32_	M_33_
4. FS_7_	M_41_	M_42_	M_43_
5. FS_3_	M_51_	M_52_	M_53_
6. FS_4_	M_61_	M_62_	M_63_
7. FS_5_	M_71_	M_72_	M_72_
8. SELECT	M_81_	M_82_	M_83_
9. Hyb_37_	M_91_	M_92_	M_93_
10. Hyb _*S*_7	M_101_	M_102_	M_103_
11. Hyb _1_7	M_111_	M_112_	M_113_
12. Ss_1_	M_121_	M_122_	M_123_
Emv8	$\sum \hat {M}_{i1},i=5,\ldots,12$	$\sum \hat {M}_{i2},i=5,\ldots,12$	$\sum \hat {M}_{i3},i=5,\ldots,12$
Ewv8	$\sum w_{i1}\hat {M}_{i1},i=5,\ldots,12$	$\sum w_{i2}\hat {M}_{i2},i=5,\ldots,12$	$\sum w_{i3}\hat {M}_{i3},i=5,\ldots,12$
Emv24	$\sum \hat {M}_{ij},i=5,\ldots,12$ and *j*=1,2,3		
Ewv24	$\sum w_{ij}\hat {M}_{ij},i=5,\ldots,12$ and *j*=1,2,3		

## Results and discussions

### Predictive accuracy of pre-miRNA classifiers by species

**Fig. 1:**
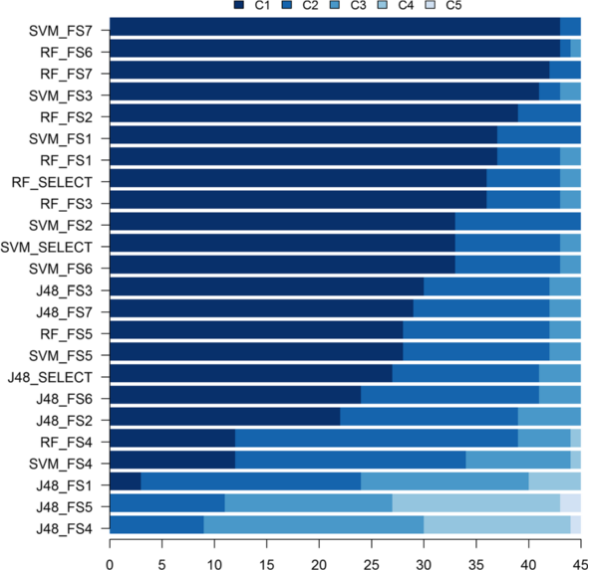
Frequencies of species for who each classification model achieved accuracies in the clusters C_1_-C_5_. $\text {Mean}_{\mathrm {C}_{1}}\ge.\,. \ge \text {Mean}_{\mathrm {C}_{5}}$

As the *F*-test on the effect of *MS* in Eq. 1 was highly significant (*p*<0.001), the effect of the simple factor *M* was studied within fixed levels of *S* (M/S_*j*_,*j*=1,…,45), and vice-verse (S/M_*l*_,*l*=1,…,24). The analysis of M/S_*j*_,*j*=1,…,45, is summarized in Fig. 1 and Table 3. The green bars in Fig. 1 indicate the pre-miRNA classifiers whose accuracy is within the cluster of maximal accuracies *C*_1_. As indicated in Fig. 1, SVMs and RFs obtained using the feature sets FS_3_, FS_6_, FS_7_ and SELECT achieved accuracies within *C*_1_ for most species. These results agree with the results reported in [12], which used larger training and test sets of human instances.

**Table 3:** Centers of accuracy clusters from 24 classification models, per species. Range = Maximum - minimum

Acronym for species	*C*_1_	*C*_2_	*C*_3_	*C*_4_	*C*_5_	Range
bfl	94	83	-	-	-	15.0
cin	83	79	75	68	-	19.0
cbr	93	85	79	-	-	17.0
cel	92	87	81	75	-	20.0
aae	95	90	80	-	-	18.0
ame	85	78	72	-	-	20.0
api	92	88	82	73	-	22.0
bmo	84	79	71	57	-	31.0
dme	91	78	-	-	-	22.0
tca	89	82	76	-	-	18.0
aca	93	86	80	-	-	16.0
xtr	97	87	82	-	-	18.0
gga	95	90	85	76	68	27.0
cfa	91	83	75	-	-	22.0
eca	93	86	77	-	-	20.0
mdo	87	79	71	-	-	21.0
mml	89	82	75	-	-	17.0
ggo	89	77	66	-	-	27.0
hsa	88	77	-	-	-	16.0
ptr	89	82	73	-	-	23.0
oan	88	83	77	70	-	23.0
cgr	92	88	84	78	-	16.0
mmu	85	79	72	-	-	17.0
rno	93	88	81	-	-	17.0
bta	84	80	75	68	-	18.0
oar	91	86	77	-	-	18.0
ssc	90	85	79	64	-	29.0
dre	93	86	80	-	-	17.0
ola	92	88	80	68	-	26.0
ppt	93	84	76	-	-	20.0
aly	95	88	81	-	-	17.0
ath	94	83	-	-	-	15.0
mes	98	91	85	-	-	14.0
gma	91	86	79	-	-	18.0
mtr	86	82	72	-	-	21.0
lus	97	84	-	-	-	18.0
mdm	98	85	-	-	-	15.0
ppe	95	87	80	-	-	18.0
ptc	94	83	-	-	-	16.0
stu	93	87	82	-	-	16.0
vvi	93	86	78	-	-	20.0
bdi	91	87	75	-	-	22.0
osa	87	77	-	-	-	16.0
sbi	96	89	81	-	-	20.0
zma	96	82	-	-	-	17.0

Figure 1 indicates only the algorithms and feature set combinations more likely to produce pre-miRNAs classifiers of maximal accuracy, but the maximal depends on the species, as it can be observed in Table 3. According to this table, the mean accuracy in *C*_1_ varied from 86 % (cin) to 96 % (ssc). As the clusters were obtained for each species using the estimated accuracies of the same 24 classifiers and the number of clusters varied from two (bfl, dme, hsa, ath, lus, mdm, ptc, osa, zma) to five (gga), Table 3 indicates that either the instances from some species are easier to classify than instances from other species, or that pre-miRNAs of different species carry specific features that identify related characteristics. In both cases, these results indicate that the incorporation of intrinsic characteristics of the species could improve the accuracy of pre-miRNAs predictive models in the classification of sequences from different species.

Table 4 presents the results of the analyzes of S/M_*l*_,*l*=1,…,24. Similar to what was observed in the analyzes of M/S_*j*_,*j*=1,…,45, the number of clusters and the corresponding centers depended on the levels of *M*. However, the number of clusters and the accuracy intervals (Range columns) in both tables show that the effect of *S* in the accuracy of pre-miRNA classifiers is broaden than the effect of *M*. For example, the number of clusters in Table 4 varied from two to six and the ranges varied from 14 % (FS_7_-RFs) to 41 % (FS_1_-J48). Moreover, although the average accuracies estimated from 17 out of 24 pre-miRNA classifiers were above 95 % for some species (column *c*_1_), the average accuracies of the same level *M*_*i*_ for other species were as low as 57 %. In fact, no *M*_*l*_,*l*=1,…,24 led to classifiers of accuracies within *c*_1_ for all species, supporting again the conjecture that the learning complexity of pre-miRNAs is species-dependent.

**Table 4:** Centers of accuracy clusters obtained from classification models induced with examples from different species, per combination of feature set and learning algorithm. Range = Maximum - minimum

Feature set	Algorithm	*c*_1_	*c*_2_	*c*_3_	*c*_4_	*c*_5_	*c*_6_	Range
FS1	SVM	95	88	78	-	-	-	21
FS2		96	92	87	80	-	-	20
FS3		95	90	85	-	-	-	15
FS4		92	86	81	77	-	-	22
FS5		94	90	86	80	-	-	20
FS6		93	88	83	-	-	-	17
FS7		95	88	-	-	-	-	16
SELECT		96	92	86	80	-	-	20
FS1	RF	97	92	87	82	72	-	30
FS2		97	93	89	83	-	-	20
FS3		95	88	84	-	-	-	18
FS4		91	87	84	79	-	-	18
FS5		92	85	77	-	-	-	19
FS6		95	88	-	-	-	-	16
FS7		96	89	-	-	-	-	14
SELECT	J48	96	92	86	78	-	-	21
FS1		98	91	85	75	67	57	41
FS2		96	90	84	77	-	-	24
FS3		97	92	87	81	-	-	21
FS4		84	79	75	69	-	-	21
FS5		83	78	75	71	-	-	17
FS6		97	93	89	83	78	72	27
FS7		96	91	87	81	-	-	21
SELECT		97	92	86	80	74	-	26

In the next subsection, we discuss how representative the instances from the 45 species considered in this work are for the induction of classifiers able to predict the classes of each other’s instances, given a classification algorithm and a feature set. In addition, we discuss the occurrence of species-specific features and their effect in the predictive accuracy of cross-species pre-miRNAs classifiers.

### Cross-species pre-miRNAs classifiers: M_*l*_ × T

Given a learning algorithm and a feature set, the relevance of the instances of a species *i* (training species) in the prediction of instances from a species *j* (test species), *i*≠*j*, can be inferred from the effects of the factors in Eq. 2. Since the *F*-test on the interaction *M*_*l*_*T* was significant (*p*≤0.05), the factor *M*_*l*_ was analyzed within each level of the factor *T* (*M*_*l*_/*T*_*j*_,*j*=1,…,45), and vise-verse (*T*/*M*_*li*_,*i*=1,…,45). The results of the analyzes of *M*_*l*_/*T*_*j*_,*j*=1,…,45 indicate the training species that resulted in pre-miRNA classifiers of higher accuracies (c_1_) for each test species. From the results of the analyzes of *T*/*M*_*li*_,*i*=1,…,45, we discussed the learning complexity of pre-miRNAs from the 45 species.

#### Choosing the training species - M_*l*_/T

**Fig. 2:**
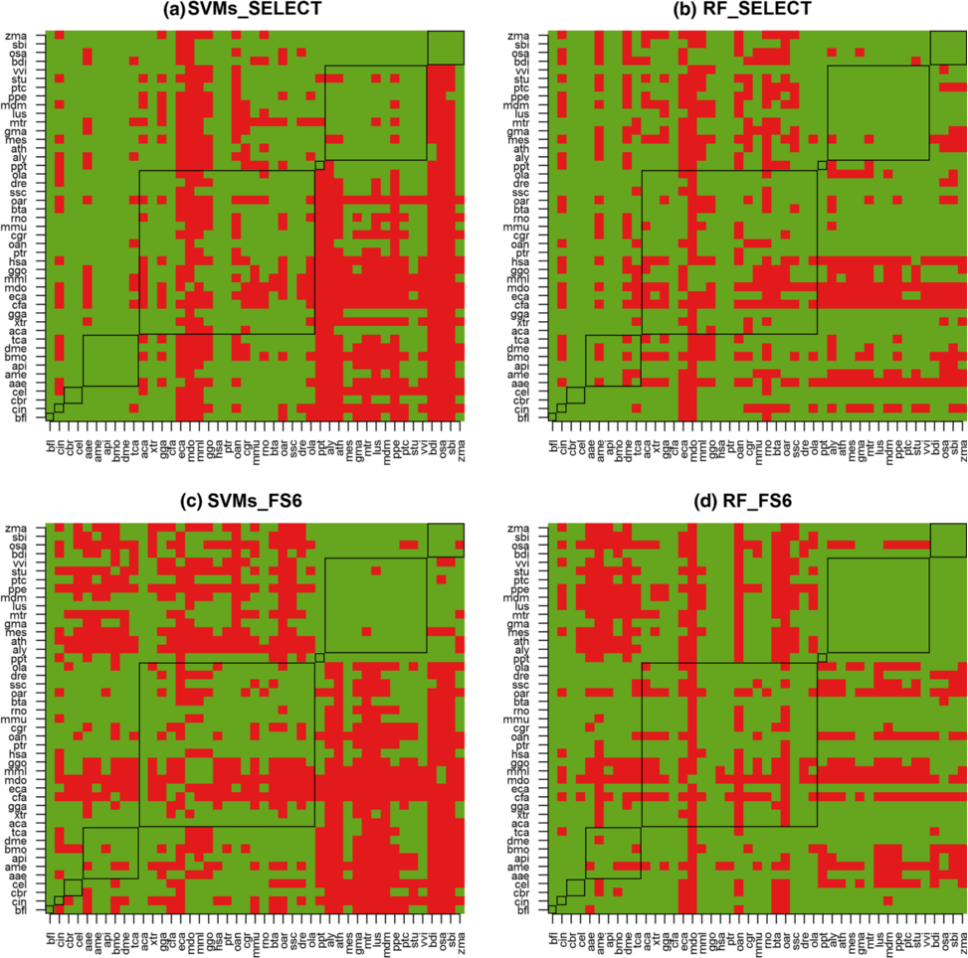
Accuracy cluster membership (columns) for cross-species pre-miRNAs classifiers. *Green* = c_1_; *red* =other; *y*-axis =model species; *x*-axis =test species; black frames encloses species from the same subphylum/class. Figures (**a**), (**b**), (**c**) and (**d**) show interactions between the learning algorithms SVMs and RF and the feature sets SELECT and FS_6_

By clustering the average accuracies *¯A*_*lij*·_, within *j*, *i, j*=1,…,45, we identified the training species *i* that led to accuracies within c_1_ for each test species *j*. Figure 2 shows these cases in green (c_1_) and red (c_2_,…,c_6_), where *i* is shown in the *Y*-axis and *j* in the *X*-axis. The results for the other 20 models were similar. As the black frames enclose species from the same subphylum/class and within each frame the green pixels are more numerous than the red ones, we conclude that a pre-miRNAs classifier was more likely to achieve predictive accuracies within c_1_ when the species *i* and *j* were from the same subphylum/class. In particular, all means *¯A*_*lij*·_ were in c_1_ when *i*=*j* (diagonal), indicating that species-specific classifiers is a good approach to improve the predictive accuracy of pre-miRNAs predictive models.

Figure 2 also shows that instances from some species were systematically harder to classify than instances from other species, which can be inferred through the number of red pixels per column. Among them, instances from mdo were typically harder to classify than instances from other species. The columns showing the clusters associated with different training species in the classification of instances from M. domestica (mdo) and L. usitatissimum (lus) illustrate these cases. Particularly, the average of the clusters obtained from SVMs_SELECT classifiers generated with instances of all species in predicting the classes of mdo instances were 80 % (c_1_), 70 % (c_2_) and 65 % (c_3_), whereas the corresponding measures for lus were 98 % (c_1_), 93 % (c_2_), 89 % (c_3_), 80 % (c_4_) and 65 % (c_5_).

Although the phylogenetic proximity of training and test species is fundamental to obtain pre-miRNAs classifiers of higher accuracies, the learning biases of the classification algorithm may increase or decrease the relevance of the subphylum/class membership, as Fig. 2 shows. In this figure, SVMs were more sensitive to the phylogenetic proximity of training and test species. While this pattern can be seen as an SVMs drawback for this problem, the phylogeny of 26 metazoan species in Additional file 3: Figure S1, shows that the distances between these species vary widely. As it can be observed in this figure, lighter areas (higher accuracies) were more frequent when training species (dendogram) and test species (rows of the matrix) were within the two groups defined by the last level of the hierarchy; one group has Hexapoda and Nematoda species and the other has Urochordata (cin) and Vertebrata species. However, the figure does not suggest a strong correlation between phylogenetic proximity and predictive accuracies when SVMs were used.

#### Inferring learning complexity - T/M_*l*_

**Fig. 3:**
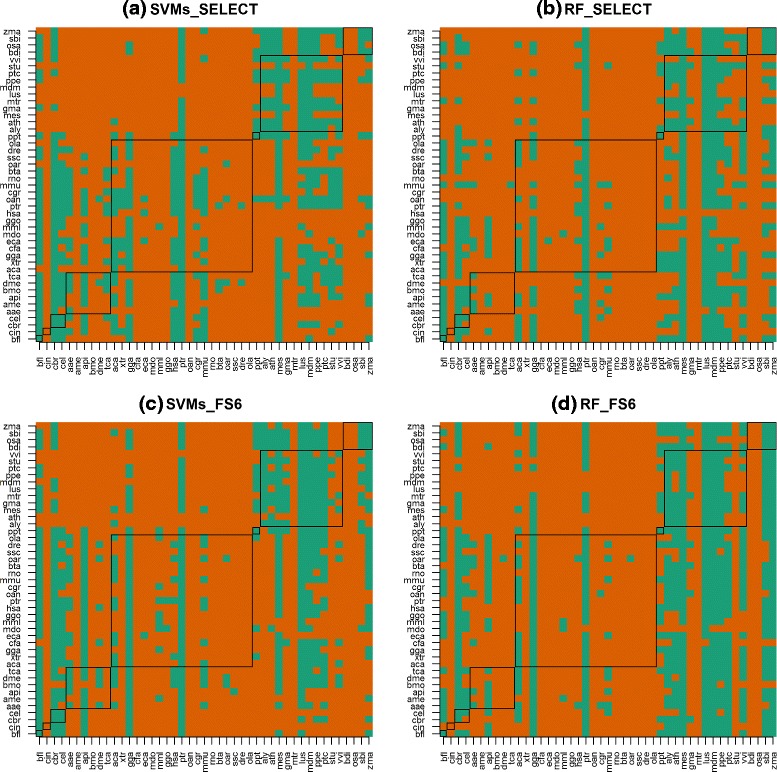
Accuracy cluster membership (*rows*) for cross-species pre-miRNAs classifiers. *Green* = c_1_; *red* =other; *y*-axis =model species; *x*-axis =test species; black frames encloses species from the same subphylum/class. Figures (**a**), (**b**), (**c**) and (**d**) show interactions between the learning algorithms SVMs and RF and the feature sets SELECT and FS_6_

In these comparisons, we clustered the accuracies estimated from all test sets, fixing the training species and a level of *M*. These clusters are displayed in Fig. 3, for four levels of *M*. In this figure, a row shows the test species (*X*-axis) assigned to the cluster *c*_1_ (green) or to another cluster (orange), when its instances were classified using a training species *i* (*Y*-axis). The highest quantities of green pixels clearly associated with the Angiosperm test species suggest that instances from Angiosperm test species were easier to classify than instances from other test species, particularly vertebrates.

Although this pattern was consistent in all 24 level of *M*, we also looked into the learning complexity by analyzing the importance of the 85 unique features in the classification of instances from all species. The idea was to indirectly compare the similarities between the instances from different species, using a feature importance measure obtained during the induction of RF classifiers. These results are discussed next.

### Feature importance

Given a feature set, the importance of each feature for the correct classification of the test set instances can be estimated by a feature importance measure, which in this work was taken from the RF results. The rationale of investigating the relevance of the RNA features used in this work for the correct classification of pre-miRNAs of different species is to infer, at least indirectly, if the phylogenetic proximity of these species is a valid criterion to choose a feature set.

The feature importance measure (*FI*) used in this study estimates the increase of misclassified OOB (Out-Of-Bag) instances when that feature is permuted in the training vectors. Since that measure is an absolute value, to allow its comparison for different classifiers induced with instances of different species, its values were re-scaled to the interval [0, 100] by the formula *RFI*=100∗(*FI*−*FI*_*min*_)/(*FI*_*max*_−*FI*_*min*_). The maximum (*FI*_*max*_) and minimum (*FI*_*min*_) *FI* values were obtained from the set of *FI* of the features used in the training step. We estimated the *RFI* values for each of the 85 unique features considered in this work feature, eliminating the 1,300 sequence structure motifs, when they were simultaneously fed to the RF algorithm to induce pre-miRNA classifiers for each of the 45 species. The pairwise Pearson correlation coefficients between species and the *RFIs* for each species are discussed next.

#### *RFI* Pearson’s correlation coefficients throughout species

**Fig. 4:**
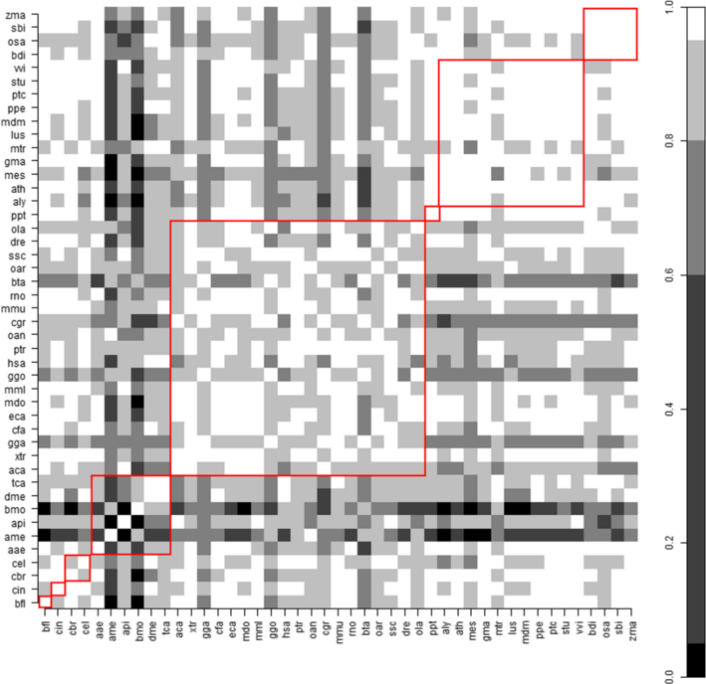
Pairwise Pearson correlation coefficient of *RFI* throughout species. Correlations breaks: 0, 0.05, 0.6, 0.8, 0.95 and 1

Figure 4 shows the pairwise Pearson correlation coefficients of *RFI* for all pairs of species. These correlations are in the interval [0, 1], where the black pixels indicate zero correlation and the white pixels indicate correlation one. Therefore, white or light gray pixels represent the cases where the pre-miRNAs of the two corresponding species shared most of the features. As the red frames indicate, these cases are more likely if the two species are from the same subphylum/class. However, there are many exceptions within and outside the subphylum/class umbrella. For example, with few exceptions (e.g. ame, bmo and bta), the features that are important for the correct classification of instances from the species bfl, cin, cbr, cel and aae, were also important for the correct classification of instances from other species. Differently, the difficulty in establishing a general rule on the association between phylogenetic proximity and feature conservation using the *RFI* criteria can be observed by the majority of dark pixels associated with Hexapoda species. This exceptions and the features with the highest *RFI* are presented next.

#### Inferring feature relevance from *RFI*

**Fig. 5:**
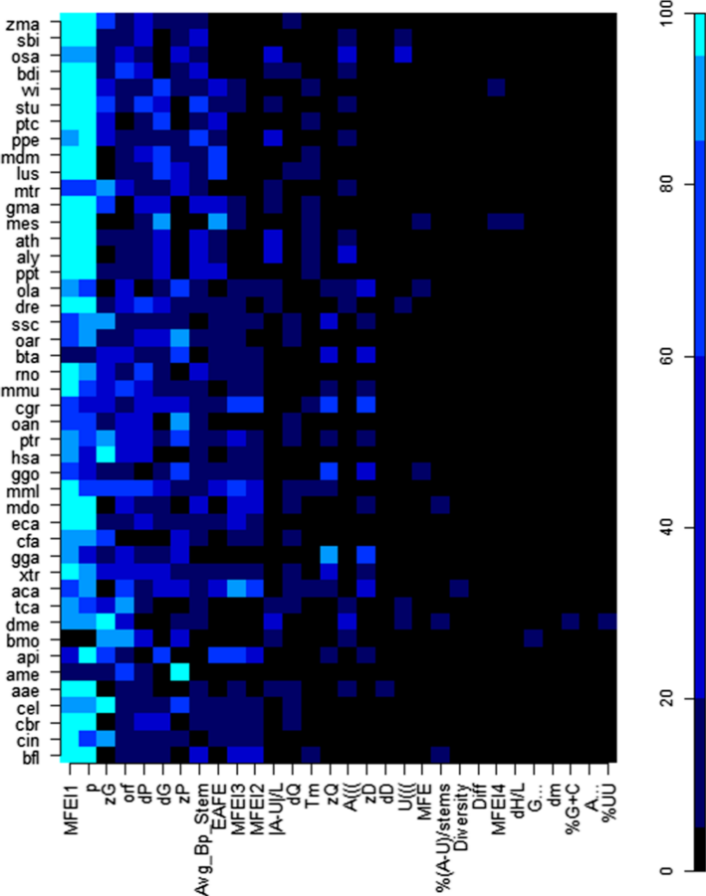
Thirty most relevant features for pre-miRNAs classification, according to *RFI* values, per species. *RFI* breaks: 0, 5, 20, 60, 85, 95 and 100

The dominance of a small number of features in the classification of human pre-miRNAs was pointed out in [12]. In that work, which used larger training sets (875+, 875-), the features that obtained the highest *FI* values were energy related (*MFEI*_1_, *zG*, *p*, *NFE*) or structural pairing patterns (*dP*, *zP*). As Fig. 5 shows, similar results were obtained in this work. The features *MFEI*_1_ and *p* were the main causes of misclassification, when permuted during the RF trainings, for most species, particularly when extracted from instances of plant species. However, those features were of lower relevance (*RFI*≤20) for the species *B. mori* (bmo), *A. mellifera* (ame) and *B. taurus* (bta). For *H. sapiens* (hsa), *D. melanogaster* (dme) and *C. elegans* (cel) instances, the feature *zG* obtained the highest *RFI*, whereas the feature of highest *RFI* for ame was *zP*.

The most interesting characteristic in Fig. 5 is the variation of *RFI* throughout species, particularly for Vertebrate and Hexapoda species. This finding suggests that no feature is prevalent in the pre-miRNAs of all species. If on one hand it can be seen as a drawback in the development of pre-miRNAs for multiple species, on the other hand, it suggests that it is possible to combine these features to obtain tools for miRNAs predictions less sensitive to characteristics inherent to different species.

### Learning biases

The small amount of highly relevant features (Fig. 5) helps to interpret the tendency of SVMs to reduce the predictive accuracy when the training and the test species were more distantly related, as those from Chordate and Angiosperm (Fig. 2). Since SVMs use the full feature space and RFs use only subspaces of it, the classification by RFs may have been dominated by features that are more conserved throughout species. The interactions between the learning biases and the species is also analyzed through the classification errors of the three learning algorithms in the next subsection.

### Classification error

**Fig. 6:**
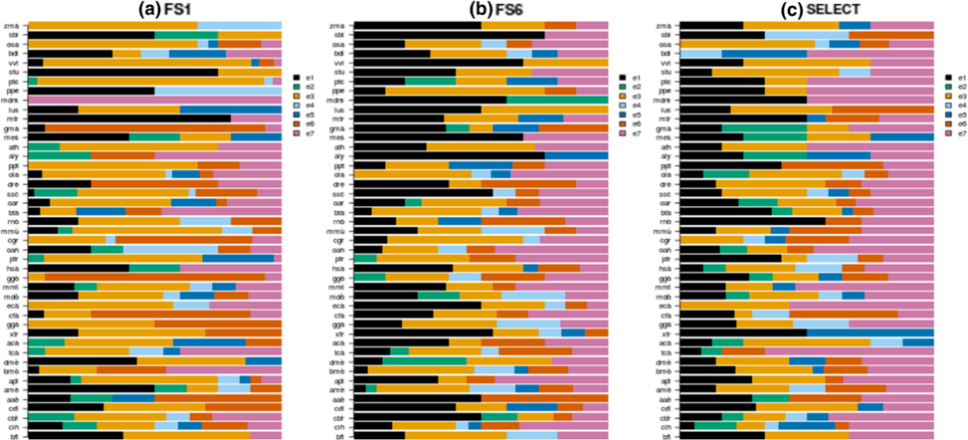
Distribution of classification errors per species. Exclusive errors by SVMs (*e*_1_), RF (*e*_2_), J48 (*e*_3_), SVMs and RF (*e*_4_), SVMs and J48 (*e*_5_), RF and J48 (*e*_6_) and SVMs and RF and J48 (*e*_7_). Figures (**a**), (**b**) and (**c**) illustrate that, fixing the feature set, the number of instances simultaneously misclassified by two or three learning algorithms depends on species

The classification errors of a particular instance by different classifiers can provide information on how typical that instance is, assuming that atypical instances or outliers are more likely to be misclassified by most classifiers. Moreover, the classification errors estimated from test sets of instances from different species by multiple classifiers is also informative of the separability of classes, in the instance space of each species. To facilitate the notation, the errors *e*_1_,*e*_2_,..,*e*_7_ are defined as exclusive classification errors of SVM (*e*_1_), RF (*e*_2_), J48 (*e*_3_), SVM and RF (*e*_4_), SVM and J48 (*e*_5_), RF and J48 (*e*_6_) and SVM and RF and J48 (*e*_7_). Since *e*_1_,…,*e*_7_ are exclusive errors, they sum one or 100 %, symbolically: $\sum _{i=1}^{7} e_{i}=1$ or $\sum _{i=1}^{7} e_{i}=100 \%$. These errors are shown in Fig. 6, for FS_1_, FS_6_ and SELECT.

As can be observed in Fig. 6, the error distributions were strongly dependent on the species, which shows in another way the classification biases associated with species sequence data. For example, Fig. 6(a) shows that *e*_1_ was zero for 15 species (cbr, tca, aca, gga, eca, ggo, ptr, cgr, ppt, aly, ath, mdm, ptc, osa, zma). Nevertheless, this same figure also shows *e*_1_ of up to 80 % for other species (e.g., bfl, cin, ame, mtr, stu, sbi). In these cases, and others where the exclusive error of a classifier induced by one of the three algorithms is higher than the errors achieved simultaneously by at least two classifiers induced by different algorithms, the separability of the classes is a matter of choosing an algorithm with the appropriate learning bias. On the other hand, the cases where *e*_7_>50*%* (e.g. mdm) could be better described by other feature spaces or by a combination of subspaces.

**Fig. 7:**
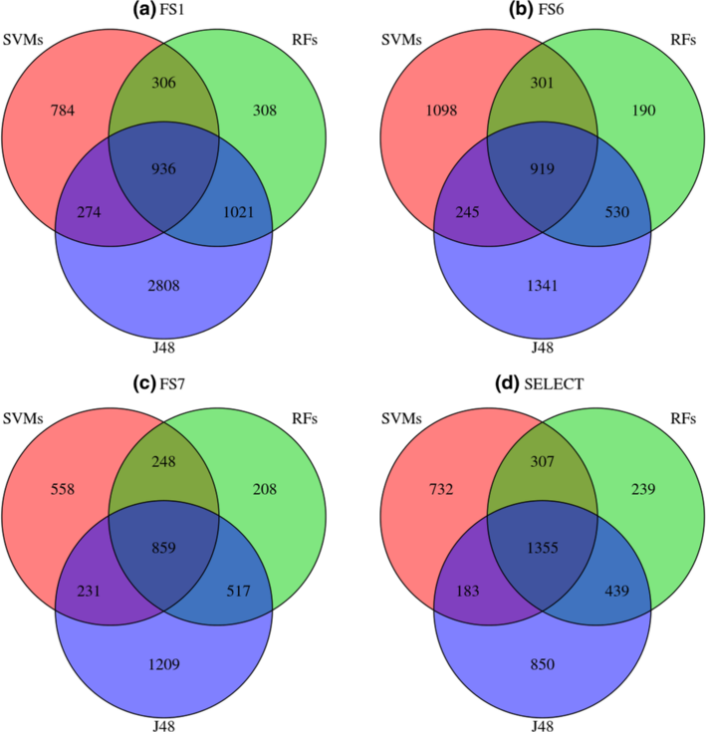
Venn diagram of the classification errors of the classification algorithms, by feature set. Results were obtained from the classification of 27,000 = 45 (test species) ×10 (repetitions) ×60 (30+,30-). Figures (**a**), (**b**), (**c**) and (**d**) illustrate that the number of instances simultaneously misclassified by two or three learning algorithms depends on the feature set

To summarize, the classification errors in each feature space, the errors *e*_1_,…,*e*_7_, were summed up for the 45 species and represented in Venn diagrams. Figure 7 shows the cases FS_1_, FS_6_, FS_7_ and SELECT. The interaction between learning algorithm and feature set, is evidenced by the large variation in the numbers of misclassified instances in the e_1_, …,e_7_ regions. For example, classification models induced by J48 tended to achieve higher exclusive error rates (*e*_3_) in higher dimensional feature spaces. Moreover, *e*_7_, the proportion of instances misclassified simultaneously by classifiers induced by the three algorithms varied by 25 % between 3.2 % and 6.7 % (3.7*%*≤*e*_7_≤6.7*%*). These two facts alone are sufficient to conjecture that the combination of multiple hypotheses may lead to pre-miRNA classifiers of higher accuracies than a single hypothesis, for a larger number of species. To provide a preliminary insight on this conjecture, we carried out additional computational experiments, using ensemble approaches to combine multiple hypothesis to improve the predictive accuracy of pre-miRNA classifiers. These results from these experiments are presented and discussed in the next subsection.

### Ensembles

Figure 7 shows the comparisons between the 44 classifiers, as defined in Table 2. According to Fig. 7, the ensembles Ewv8_SVMs, Emv8_SVMs, Emv24, Ewv24, Emv8_RF, Ewv8_RF and the classifiers obtained with the new feature sets presented better predictive accuracies than the 24 previously discussed (Fig. 1), for many species, although none of the them achieved predictive accuracies within C_1_ for all 45 species. Moreover, it is important to remind that these ensembles and the new feature sets do not include features extracted from shuffled sequences. Figure 7 also shows that the simple combination of different hypotheses can increase the predictive accuracy, even using the algorithm J48, which typically led to equal or lower classification accuracies than RFs and SVMs.

**Fig. 8:**
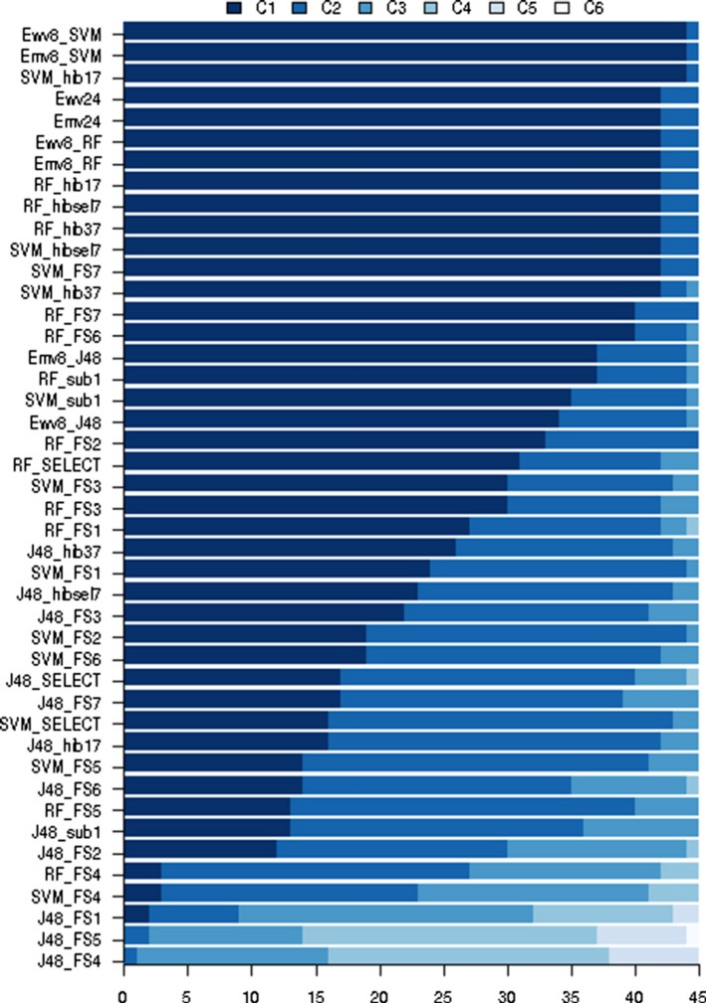
Distribution of the accuracies of 44 classifiers within the accuracy clusters. $\text {Mean}_{C_{1}} > \ldots > \text {Mean}_{C_{6}}$

Based on the results shown in Figs. 5, 7 and 8, it is possible to state that it is unlike that a unique learning algorithm and a unique set of features is able to produce the best pre-miRNA predictive model for all species. In fact, the experimental results obtained in this study suggested that the learning of good predictive models for pre-miRNAs classification depends on the learning complexity inherited of the problem and the peculiarities of the instances from different species. Since ensembles apparently provide an alternative and efficient approach to accommodate these peculiarities, an appropriate construction of hypothesis diversity (e.g. [32]) may enhance the performance of miRNA discovery tools in the classification of pre-miRNAs of different species.

#### Benchmarking

The main focus of this research was to investigate the importance of different feature sets throughout species, and not to develop a new method for predicting miRNAs. The authors believe that the results from this study will potentially help in the design of new methods, which should then be subject to benchmarking against other state of the art methods; an analysis which is not applicable to this study. Moreover, since the adopted approach compared the predictive accuracies of classifiers obtained with a feature set in different species, the computational experiments were designed to produce classifiers whose predictive accuracies differ only by the species. For example, the low availability of positive examples for many species imposed the restriction of using small equal sized training sets (60+,60-) for all species, which certainly leads to lower predictive accuracies. Nevertheless, the ensembles evaluated in this work were benchmarked against 36 single classifiers, from which seven feature sets and algorithms combination are implemented in seven tools from the literature.

Finally, the scripts necessary scripts to reproduce the results presented in this work are publicly available from: http://dx.doi.org/10.5281/zenodo.49754.

## Conclusion

The increase in sequencing capacity and the computational analysis of large amounts of sequencing data to detect miRNAs supported the recent advances in the discovery of novel miRNAs from over a hundred species. Albeit miRNA systems vary throughout species, miRNA discovery tools from the literature have not addressed the impact of these differences. As a consequence, the performance of these tools is usually reduced when data sets from species not used in their development are analyzed. Building species-specific miRNA discovery tools may not be always viable, for example for lack of training data. Since the detection of putative pre-miRNAs is an important step in the development of miRNA discovery tools, it is important to investigate how the peculiarities naturally occurring in pre-miRNAs between species relate to the learning bias of machine learning approaches. In this study, we presented the results of a systematic investigation on the automatic learning of pre-miRNAs of 45 species, using techniques traditionally employed by miRNA discovery tools from the literature. The results presented in this study not only showed the need to develop new approaches to handle the intrinsic characteristics of pre-miRNAs from different species, but we also indicated one potential way to go forward, using ensemble methods built with computationally efficient features.

## Additional files

Additional file 1 Phylum/division, subphylum/class, species, acronyms, number of positive examples available at miRBase 20, mean and standard deviation of the length distributions. NR=Non-Redundant. (PDF 17.8 kb)

Additional file 2 Phylum/division, subphylum/class, species, acronyms, number of redundant negative examples out of 1,000 sequences excised from CDS or pseudo genes and the corresponding website link for download. (PDF 27.1 kb)

Additional file 3 Phylogeny and predictive accuracy of 26 metazoan species. Phylogeny extracted from the phylogenetic tree in TreeFam (http://www.treefam.org/browse#tabview=tab2) [36]. (JPG 719 kb)
