# Supplementary material for: Automatic learning of pre-miRNAs from different species
Source: BMC Bioinformatics. 2016 May 28;17:224. doi: 10.1186/s12859-016-1036-3 (PMC4884428; doi:10.1186/s12859-016-1036-3)
Supplement: Additional file 1 — Phylum/division, subphylum/class, species, acronyms, number of positive examples available at miRBase 20, mean and standard deviation of the length distributions. NR=Non-Redundant. (PDF 17.8 kb) [file 12859_2016_1036_MOESM1_ESM.pdf]

Phylum/Division	Subphylum/Class	Species genus	Acronym	#pre-miRNA		Length
				All	NR	(Mean ± SD)
Chordate	Cephalochordata	Branchiostoma floridae	bfl	156	143	87 ± 13
	Urochordata	Ciona intestinalis	cin	346	331	63 ± 16
	Nematoda	Caenorhabditis briggsae	cbr	177	148	92 ± 19
		Caenorhabditis elegans	cel	233	214	89 ± 17
	Hexapoda	Aedes aegypti	aae	101	90	94 ± 21
		Apis mellifera	ame	218	215	100 ± 20
		Acyrthosiphon pisum	api	117	101	66 ± 9
		Bombyx mori	bmo	489	432	100 ± 22
		Drosophila melanogaster	dme	238	236	95 ± 23
		Tribolium castaneum	tca	220	210	95 ± 22
	Vertebrate	Anolis carolinensis	aca	282	272	89 ± 9
		Xenopus tropicalis	xtr	189	163	83 ± 11
		Gallus gallus	gga	734	695	92 ± 17
		Canis familiaris	cfa	324	280	69 ± 14
		Equus caballus	eca	341	298	78 ± 15
		Monodelphis domestica	mdo	460	370	67 ± 12
		Macaca mulatta	mml	615	524	86 ± 17
		Gorilla gorilla	ggo	332	313	105 ± 12
		Homo sapiens	hsa	1,872	1,640	82 ± 17
		Pan troglodytes	ptr	659	542	90 ± 17
		Ornithorhynchus anatinus	oan	396	327	100 ± 24
		Cricetulus griseus	cgr	200	199	82 ± 12
		Mus musculus	mmu	1,186	1,078	83 ± 19
		Rattus norvegicus	rno	449	428	92 ± 17
		Bos taurus	bta	798	710	80 ± 13
		Ovis aries	oar	105	96	97 ± 18
		Sus scrofa	ssc	280	247	81 ± 10
		Danio rerio	dre	346	240	93 ± 18
		Oryzias latipes	ola	168	146	95 ± 9
Bryophyta	Musci	Physcomitrella patens	ppt	229	204	161 ± 56
Angiospermae	Eudicotyledons	Arabidopsis lyrata	aly	298	177	183 ± 100
		Arabidopsis thaliana	ath	298	257	183 ± 103
		Manihot esculenta	mes	153	109	117 ± 38
		Glycine max	gma	505	361	131 ± 47
		Medicago truncatula	mtr	672	373	165 ± 91
		Linum usitatissimum	lus	124	100	144 ± 34
		Malus domestica	mdm	206	90	130 ± 66
		Prunus persica	ppe	180	147	136 ± 51
		Populus trichocarpa	ptc	352	246	128 ± 46
		Solanum tuberosum	stu	224	163	95 ± 43
		Vitis vinifera	vvi	163	131	127 ± 56
		Brachypodium distachyon	bdi	258	228	178 ± 101
	Monocotyledons	Oryza sativa	osa	592	482	153 ± 77
		Sorghum bicolor	sbi	205	174	142 ± 54
		Zea mays	zma	172	133	132 ± 45
