# Supplementary material for: Automatic learning of pre-miRNAs from different species
Source: BMC Bioinformatics. 2016 May 28;17:224. doi: 10.1186/s12859-016-1036-3 (PMC4884428; doi:10.1186/s12859-016-1036-3)
Supplement: Additional file 2 — Phylum/division, subphylum/class, species, acronyms, number of redundant negative examples out of 1,000 sequences excised from CDS or pseudo genes and the corresponding website link for download. (PDF 27.1 kb) [file 12859_2016_1036_MOESM2_ESM.pdf]

Phylum/Division	Subphylum/Class	Species genus	Acronym	#Redundant	Web Source
Chordate	Cephalochordata	Branchiostoma floridae	bfl	1	Metazome v3.0
	Urochordata	Ciona intestinalis	cin	3	Metazome v3.0
	Nematoda	Caenorhabditis briggsae	cbr	7	Metazome v3.0
		Caenorhabditis elegans	cel	1	Metazome v3.0
	Hexapoda	Aedes aegypti	aae	4	Metazome v3.0
		Apis mellifera	ame	395	NCBI
		Acyrthosiphon pisum	api	43	NCBI
		Bombyx mori	bmo	5	Metazome v3.0
		Drosophila melanogaster	dme	3	Metazome v3.0
		Tribolium castaneum	tca	0	Metazome v3.0
	Vertebrate	Anolis carolinensis	aca	35	NCBI
		Xenopus tropicalis	xtr	2	Metazome v3.0
		Gallus gallus	gga	3	Metazome v3.0
		Canis familiaris	cfa	4	Metazome v3.0
		Equus caballus	eca	14	NCBI
		Monodelphis domestica	mdo	8	Metazome v3.0
		Macaca mulatta	mml	80	NCBI
		Gorilla gorilla	ggo	61	NCBI
		Homo sapiens	hsa	3	Metazome v3.0
		Pan troglodytes	ptr	88	NCBI
		Ornithorhynchus anatinus	oan	50	NCBI
		Cricetulus griseus	cgr	12	NCBI
		Mus musculus	mmu	5	Metazome v3.0
		Rattus norvegicus	rno	3	Metazome v3.0
		Bos taurus	bta	94	NCBI
		Ovis aries	oar	70	NCBI
		Sus scrofa	ssc	62	NCBI
		Danio rerio	dre	3	Metazome v3.0
		Oryzias latipes	ola	0	Metazome v3.0
Bryophyta	Musci	Physcomitrella patens	ppt	3	Phytozome v9.0
Angiospermae	Eudicotyledons	Arabidopsis lyrata	aly	5	Phytozome v9.0
		Arabidopsis thaliana	ath	4	Phytozome v9.0
		Manihot esculenta	mes	3	Phytozome v9.0
		Glycine max	gma	4	Phytozome v9.0
		Medicago truncatula	mtr	2	Phytozome v9.0
		Linum usitatissimum	lus	2	Phytozome v9.0
		Malus domestica	mdm	3	Phytozome v9.0
		Prunus persica	ppe	15	Phytozome v9.0
		Populus trichocarpa	ptc	3	Phytozome v9.0
		Solanum tuberosum	stu	2	Phytozome v9.0
		Vitis vinifera	vvi	0	Phytozome v9.0
		Brachypodium distachyon	bdi	2	Phytozome v9.0
	Monocotyledons	Oryza sativa	osa	4	Phytozome v9.0
		Sorghum bicolor	sbi	3	Phytozome v9.0
		Zea mays	zma	1	Phytozome v9.0
