# Supplementary figures and images for: Automatic learning of pre-miRNAs from different species
Source: BMC Bioinformatics. 2016 May 28;17:224. doi: 10.1186/s12859-016-1036-3 (PMC4884428; doi:10.1186/s12859-016-1036-3)

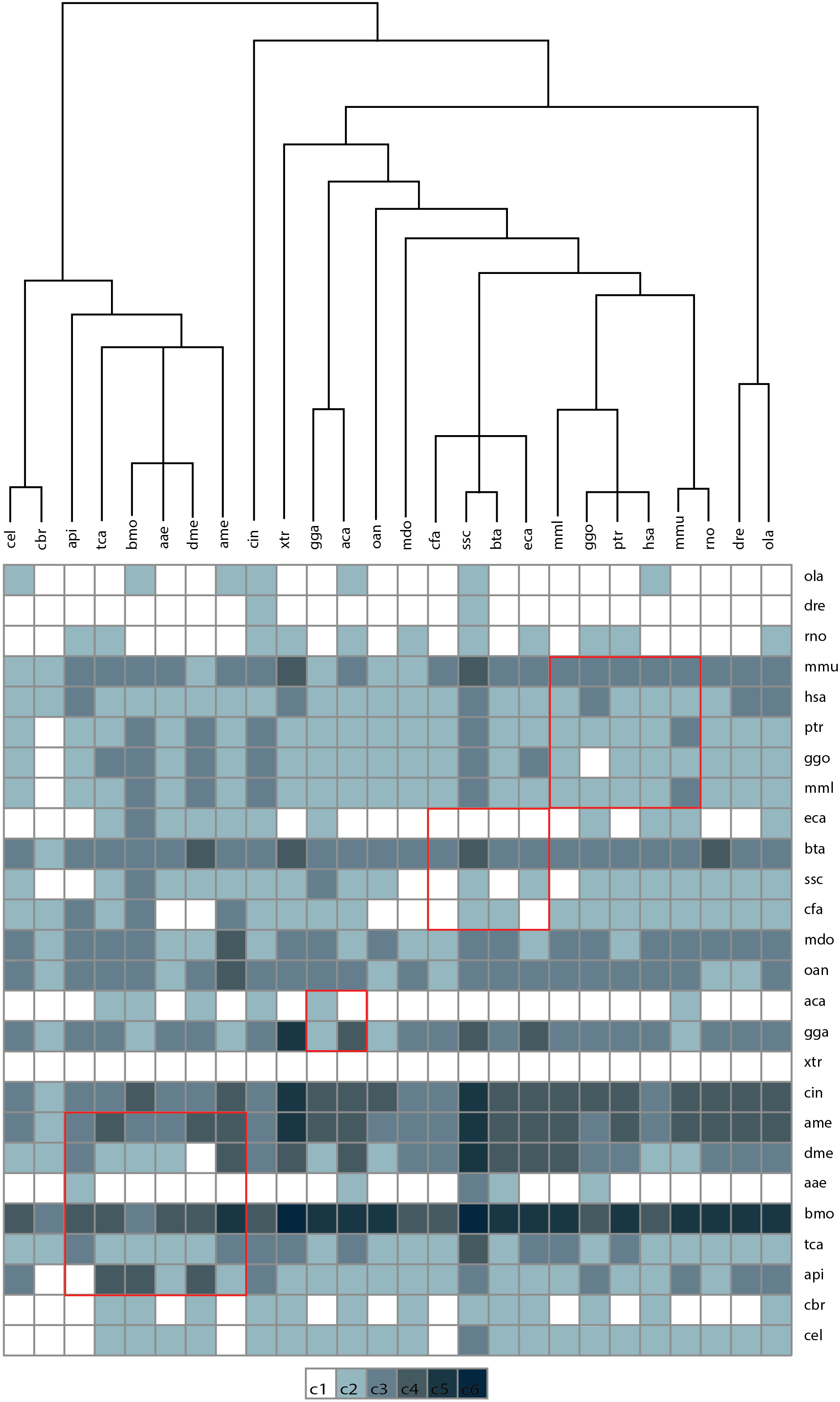

Supplement: Additional file 3 — Phylogeny and predictive accuracy of 26 metazoan species. Phylogeny extracted from the phylogenetic tree in TreeFam (http://www.treefam.org/browse#tabview=tab2) [36]. (JPG 719 kb) [file 12859_2016_1036_MOESM3_ESM.jpg]
